# Effect of DNA Methylation in Various Diseases and the Probable Protective Role of Nutrition: A Mini-Review

**DOI:** 10.7759/cureus.309

**Published:** 2015-08-24

**Authors:** Venkataramana Kandi, Sabitha Vadakedath

**Affiliations:** 1 Department of Microbiology, Prathima Institute of Medical Sciences; 2 Biochemistry, Chalmeda Anand Rao Institute of Medical Sciences

**Keywords:** dna methylation, epigenetic modifications, imprinting disorders, methyltransferase

## Abstract

DNA methylation, a process of adding a methyl group to DNA done by a DNA methyltransferase is a heritable (epigenetic) alteration leading to cancer, atherosclerosis, nervous disorders (Imprinting disorders), and cardiovascular diseases. The role of nutrition in DNA methylation is revealed by identification of methyl variable positions (MVP) on DNA. These regions are more susceptible to DNA methylations. Nutritional supplementation of folic acid and methionine in utero and in adults decreased epigenetic modifications due to its role in DNA metabolism (one carbon metabolism). Thus, in utero and adult supplementation of folic acid and methionine may reduce DNA methylation. This review attempts to highlight the process of DNA methylation, its effect on various diseases, and the probable protective role of nutrition.

## Introduction and background

**Figure 1:**
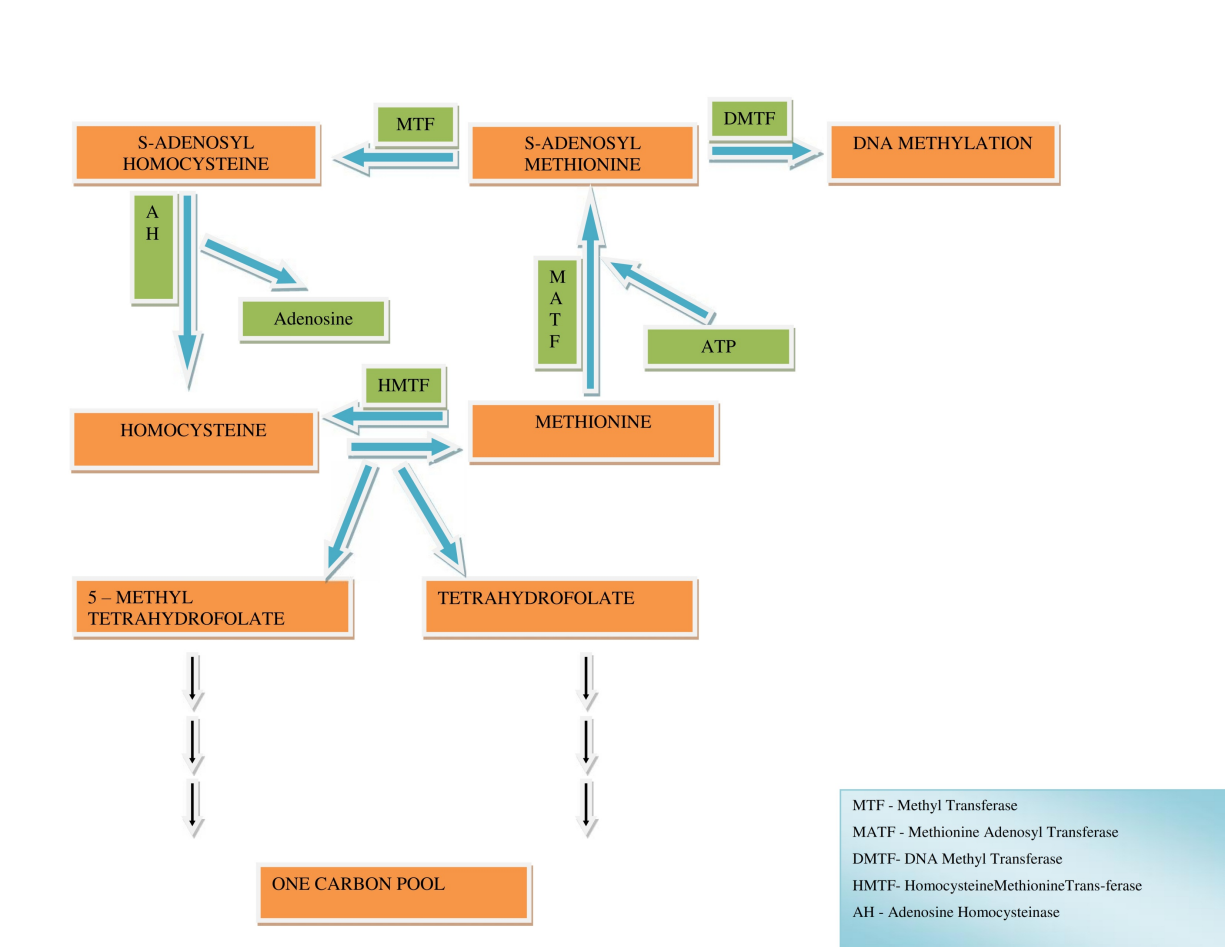
Reactions contributing to DNA methylation

DNA methylation is a genetic process that is extensively being researched among mammals, including humans. The epigenetic modifications in DNA contribute to the regulation of gene expression. DNA methylation is associated with histone modifications, which play a key role in regulating the functioning of the DNA by altering chromatin structure. The information imprinted on the genes is epigenetically marked during gametogenesis and is expressed/transferred to the offspring's familially. The process of genetic transfer may be influenced by a phenomenon called as DNA methylation. The significance of DNA methylation and its role in the development of various diseases/processes that include formation of tumours, atherosclerosis, cardiovascular diseases, imprinting disorders, and ageing is least understood. Considering the fact that DNA methylation is a reversible process, we have attempted to highlight the effect of DNA methylation in various diseases and the beneficial role of nutrition in this mini review. The process of addition of the methyl group to DNA is DNA methylation. The major donor of the methyl group is s-adenosylmethionine (SAM) formed from methionine, and the methylation reaction is done by the action of the enzyme DNA methyltransferase (DMT) as shown in Figure 1 [1].

The methyl group is accepted by either adenine or cytosine of DNA. Adenine methylation is predominant in prokaryotes, whereas cytosine methylation is dominant in eukaryotes with variability in different species [2].

## Review

### DNA methyltransferase (DMTF)

DNA methyltransferase (DMTF) is an enzyme involved in methylating cytosine/adenine residues of DNA. There are two types of DMTF: the maintenance DMTF and the de novo DMTF [3]. Maintenance DMTF helps in copying methylated patterns of parent strands to the daughter strand during the replication process. De novo DMTF helps in creating new methylated patterns to DNA during development [4]. In stem cells, the non-methylated regions of DNA are lost during cell differentiation and are a specific feature of stem cells when compared with fibroblasts. Aberrant DNA methylation might contribute to decreased transcriptional activity of some genes involved in stem cell maintenance and differentiation. Thus, methylated/non-methylated DNA pattern transmission depends completely on their location within the gene [5].

### Effects of DNA methylation

DNA methylation is an epigenetic modification, i.e., heritable change in DNA without any modifications to the sequence of DNA. It alters expression of a gene during cell differentiation and causes a change that is heritable. Methylated modifications of DNA occur during the mitotic or meiotic division of the cell [6]. Methylated DNA usually represses transcription of a gene and these methylated DNA are not recognized by RNA polymerases; thus, it becomes one of the mechanisms to control gene expression [7]. DNA methylation in turn regulates the expression of enzymes, like histone deacetylase, resulting in the modification of chromatin structure [8].

DNA methylation changes are associated with a number of diseases such as imprinting disorders [9], cardiovascular diseases [10], autoimmune diseases, neurological disorders [11], and cancer [12]. The advantages of DNA methylation in humans include regulation of long-term storage of memory, which can be used to estimate age, and biological clocks in humans [13-15]. DNA can be demethylated by the base-excision repair system.

### Cancer

In general, altered DNA methylation is an important factor associated with cancer development. Hypermethylation in an abnormal state leads to transcriptional silencing and gene inactivation, whereas hypomethylation is linked to chromosomal instability and loss of imprinting (transfer of methylated patterns to daughter cells). Thus, DNA methylation can cause hypermethylation of tumor suppressor genes (oncogene suppressor) and hypomethylation of oncogenes [16-17].

### Imprinting disorders

The process of expressing the DNA methylation pattern of a specific gene based on parental origin is known as imprinting [18]. The failure of this process of imprinting leads to imprinting disorders. Angelman’s syndrome is an imprinted neurodevelopmental disorder caused due to the deficiency of maternal ubiquitin ligase enzyme. Prader–Willi syndrome is also an imprinting neurobehavourial disorder due to the deficiency of ubiquitin ligase enzyme of paternal origin. Thus, loss of the maternal/paternal imprinting centre leads to abnormal DNA methylations, which could lead to epigenetic modification in DNA [19-20]. Loss of imprinting not only in DNA methylation but also in the binding protein involved in methylation also contributes to imprinting disorders.

### Aging

DNA methylation is suppressed during zygote formation and enhanced methylation is seen during development. There is a loss of DNA methylation during aging [21]. Biological clocks act as biomarkers for predicting the process of aging [22]. With the advancement in age, the genes, such as estrogen receptor, insulin–like growth factor 2 (IGF2), p^16^, etc., get hypermethylated, and there is abnormal DNA methylation leading to a heritable change.

### Atherosclerosis

**Figure 2:**
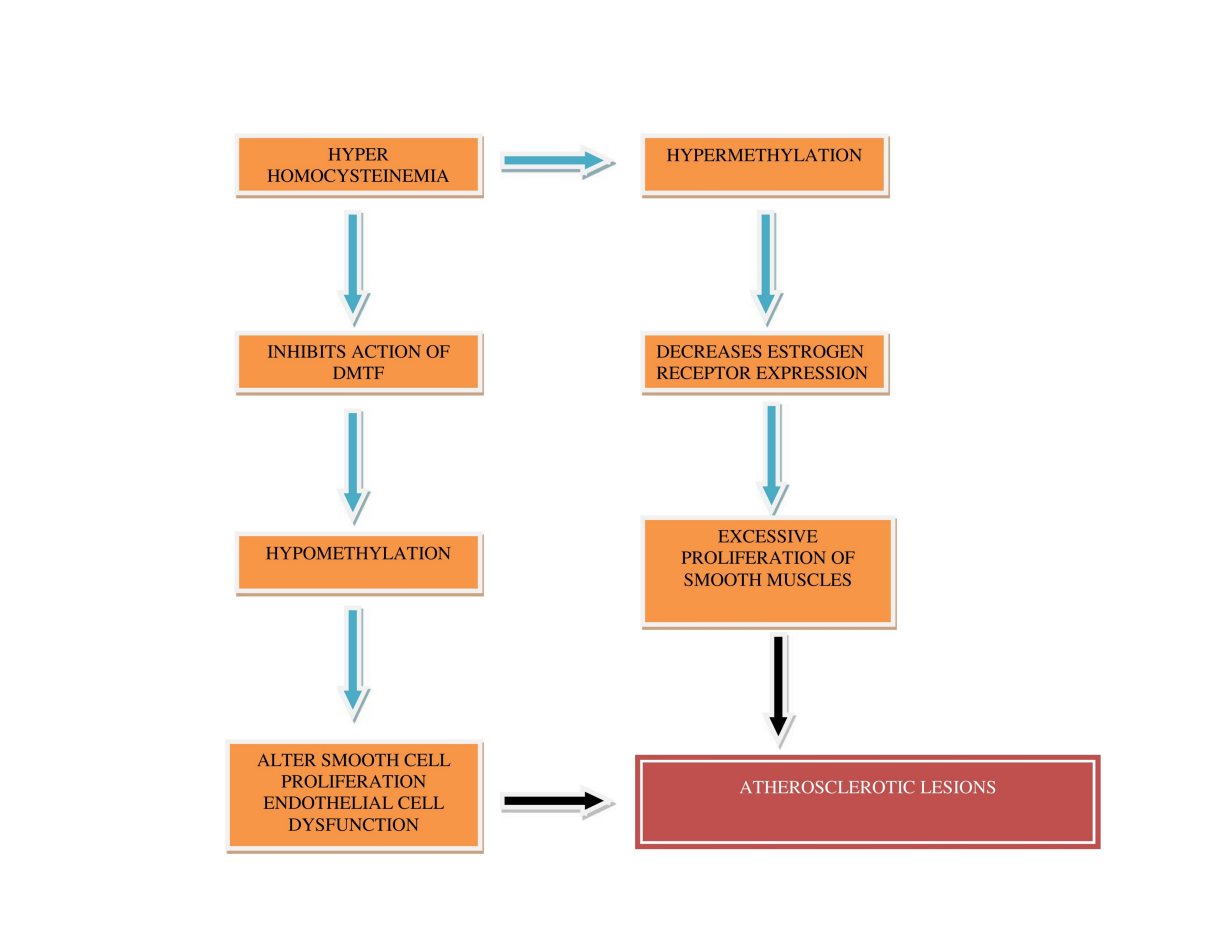
Flow chart showing various causes of hyper/hypo methylation of DNA leading to Atherosclerotic lesions

DNA methylation polymorphism forms an important biomarker for atherosclerosis. Monocytes and lymphocytes are the sites for DNA methylation polymorphism [23-25] (Figure 2).

Thus, either hypo- or hypermethylation leads to altered smooth muscle cell proliferation contributing to atherosclerotic lesions. Hypomethylation of monocytes and lymphocytes and hypermethylation of gene-specific areas takes place. Atherosclerosis, if untreated, leads to cardiovascular disease.

### Nutrition: Its role in DNA methylation

Nutrition plays an important role in health, disease conditions, and influences in utero development. There are areas on DNA, which are sensitive to methylation based on few previous studies on nutrition, and these regions are identified as methylation variable positions (MVP). The observation of MVPs explains the role of nutrition in the fetal origin of adult diseases. There are several theories put forward to explain the fetal origin of adult diseases. These theories describe that nutrition plays an important role on adult disease risk and its transfer to the offspring [26]. It also predicts that fetal growth impairment is associated with adult cardiovascular disease, diabetes, and insulin resistance [27-28]. A previous study has revealed that prenatal and early post-natal exposure to famine may increase the risk of obesity [29], schizophrenia, lung diseases, and even breast cancer in women [30].

But in humans, folic acid and methionine play an important role in DNA modifications. As we know that folic acid functions as a one-carbon metabolism that supplies carbon for purine and pyrimidine synthesis, it becomes important to have a good understanding of its role in DNA modifications both in utero and in adults. Periconceptional supplementation of folic acid increased DNA methylation by 4.5% of IGF2 [31]. Along with methyl supplementation, the folic acid requirement is very much necessary for postmenopausal women not only for proper nervous system functions but also for preventing epigenetic alterations [32]. In a study, cord blood sample analysis revealed that folic acid status is inversely related to homocysteine and vice-versa [33]. DNA methylation of 12 genes (EIF2C3, ZBTB11, BDH2, ZNF187, RUNX1T1, C9 or f64, PDE2A, MGC33486, AMN, ZPBP2, FBN3, and PVRL2) directly correlated to homocysteine levels and methylation of five genes (ATP5F1, CYP26C1, FSTL3, MDS032, and BMX) showed an inverse correlation with homocysteine levels [34]. Thus, nutrient-based DNA methylation is gene specific, site specific, tissue specific, and age specific.

## Conclusions

DNA methylation can lead to an epigenetic change, as it regulates gene expression by controlling the transcription of the concerned genes. Folic acid and methionine play a significant role in restraining DNA methylation; in utero and adult supplementation of these nutrients can prevent tumorigenesis and harmful effects of DNA methylation. Further research should concentrate on the usefulness of therapeutic interventions in minimizing the adverse effects of DNA methylation.
